# Piperazine-1,4-diium diacetate

**DOI:** 10.1107/S1600536811047441

**Published:** 2011-11-12

**Authors:** Shao-Gang Hou

**Affiliations:** a Department of Chemical & Environmental Engineering, Anyang Institute of Technology, Anyang 455000, People’s Republic of China

## Abstract

In the title salt, C_4_H_12_N_2_^2+^·2C_2_H_3_O_2_^−^, the piperazine-1,4-diium cation has 2/*m* symmetry with the NH_2_ unit located on a mirror plane and the acetate anion has *m* symmetry with all non-H atoms and one H atom located on a mirror plane. The piperazine ring adopts a chair conformation. In the crystal, the cations are linked with the anions *via* N—H⋯O hydrogen bonding into chains parallel to the *c* axis.

## Related literature

For the synthesis and properties of related compounds, see: Blagden *et al.* (2008 ▶); Vishweshwar *et al.* (2006 ▶); Fu *et al.* (2009 ▶).
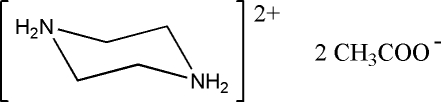


## Experimental

### 

#### Crystal data

C_4_H_12_N_2_^2+^·2C_2_H_3_O_2_^−^
*M* *_r_* = 206.24
Monoclinic, 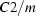

*a* = 13.1704 (1) Å
*b* = 7.1820 (2) Å
*c* = 5.7975 (5) Å
β = 101.904 (1)°
*V* = 536.59 (5) Å^3^
*Z* = 2
Mo *K*α radiation
μ = 0.10 mm^−1^
*T* = 298 K
0.30 × 0.25 × 0.15 mm

#### Data collection

Rigaku Mercury2 diffractometer
Absorption correction: multi-scan (*CrystalClear*; Rigaku, 2005 ▶) *T*_min_ = 0.90, *T*_max_ = 0.99
1396 measured reflections
647 independent reflections
582 reflections with *I* > 2σ(*I*)
*R*_int_ = 0.018

#### Refinement

*R*[*F* ^2^ > 2σ(*F* ^2^)] = 0.048
*wR*(*F* ^2^) = 0.140
*S* = 1.11
647 reflections
40 parameters
2 restraints
H-atom parameters constrained
Δρ_max_ = 0.29 e Å^−3^
Δρ_min_ = −0.26 e Å^−3^

### 

Data collection: *CrystalClear* (Rigaku, 2005 ▶); cell refinement: *CrystalClear*; data reduction: *CrystalClear*; program(s) used to solve structure: *SHELXTL* (Sheldrick, 2008 ▶); program(s) used to refine structure: *SHELXTL*; molecular graphics: *SHELXTL*; software used to prepare material for publication: *SHELXTL*.

## Supplementary Material

Crystal structure: contains datablock(s) I, global. DOI: 10.1107/S1600536811047441/xu5380sup1.cif

Structure factors: contains datablock(s) I. DOI: 10.1107/S1600536811047441/xu5380Isup2.hkl

Supplementary material file. DOI: 10.1107/S1600536811047441/xu5380Isup3.cml

Additional supplementary materials:  crystallographic information; 3D view; checkCIF report

## Figures and Tables

**Table 1:** Hydrogen-bond geometry (Å, °)

*D*—H⋯*A*	*D*—H	H⋯*A*	*D*⋯*A*	*D*—H⋯*A*
N1—H1*A*⋯O2^i^	0.90	1.80	2.694 (2)	176
N1—H1*B*⋯O1	0.90	1.79	2.680 (2)	170

Symmetry code: (i) 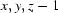.

**Fig. 1.**
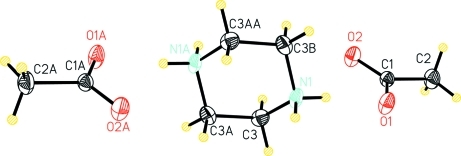
Molecular view of the title compound with the atomic numbering scheme. Displacement ellipsoids are drawn at the 30% probability level.

**Fig. 2.**
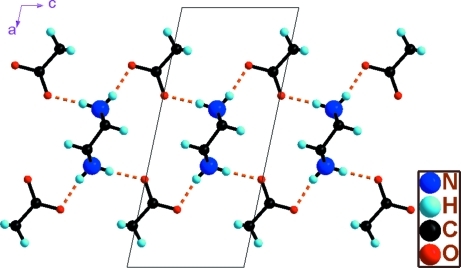
The crystal packing of the title compound viewed along the b axis showing the one-dimensionnal hydrogen bondings chain (dashed line).

## supplementary crystallographic information

### Comment

The amino derivatives have found wide range of applications in material science, such as solid crystalline materials with special optical and dielectric behaviors (Fu *et al.* 2009). With the purpose of obtaining solid crystalline materials of amino compounds, various amines have been studied and a series of new salts with this organic molecules have elaborated (Blagden *et al.* 2008; Vishweshwar, *et al.* 2006). The synthesis of organic salts often relies on the acid-amide H-bonds interactions. Herein, we report the crystal structure of the title compound, piperazine-1,4-diium acetate.

The asymmetric unit is composed of a quarter piperazine-1,4-diium cation and half acetate anion (Fig.1). The amine N1 atom was protonated. And the carboxyl group was deprotonated to keep the charge balance. The whole anion and N1 atom were located on the *ac* plane. The geometric parameters of the title compound are in the normal range.

In the crystal structure, all the amino H atoms and hydroxy H atom are involved in intermolecular N—H···O hydrogen bonds interactions with the carboxyl O atoms. These hydrogen bonds link the ionic units into a one-dimentional chain parallel to the *c*-axis (Table 1 and Fig.2).

### Experimental

A mixture of piperazine (2.0 mmol) and acetic acid (2.0 mL) in 20 mL distilled water was refluxed for 5 h, then cooled and filtrated. The filtrate was evaporated slowly in the air. Colorless block crystals suitable for X-ray analysis were obtained after one week.

### Refinement

All H atoms attached to C atoms were fixed geometrically and treated as riding with C—H = 0.97 Å (methylene) and C—H = 0.96 Å (methyl) with *U*_iso_(H) = 1.2*U*_eq_(methylene) and *U*_iso_(H) = 1.5*U*_eq_(methyl). H atoms bonded to N atoms were located in difference Fourier map and restrained with the H—N1 = 0.90 (2)Å. In the last stage of refinement they were treated as riding on the N atom with *U*_iso_(H) = 1.5*U*_eq_(N).

### Crystal data

**Table:**

C_4_H_12_N_2_^2+^·2C_2_H_3_O_2_^−^	*F*(000) = 224
*M**_r_* = 206.24	*D*_x_ = 1.276 Mg m^−^^3^
Monoclinic, *C*2/*m*	Mo *K*α radiation, λ = 0.71073 Å
Hall symbol: -C 2y	Cell parameters from 647 reflections
*a* = 13.1704 (1) Å	θ = 3.6–27.5°
*b* = 7.1820 (2) Å	µ = 0.10 mm^−^^1^
*c* = 5.7975 (5) Å	*T* = 298 K
β = 101.904 (1)°	Block, colorless
*V* = 536.59 (5) Å^3^	0.30 × 0.25 × 0.15 mm
*Z* = 2	

### Data collection

**Table:**

Rigaku Mercury2 diffractometer	647 independent reflections
Radiation source: fine-focus sealed tube	582 reflections with *I* > 2σ(*I*)
graphite	*R*_int_ = 0.018
Detector resolution: 13.6612 pixels mm^-1^	θ_max_ = 27.5°, θ_min_ = 3.6°
CCD profile fitting scans	*h* = −16→16
Absorption correction: multi-scan (*CrystalClear*; Rigaku, 2005)	*k* = −9→5
*T*_min_ = 0.90, *T*_max_ = 0.99	*l* = −7→6
1396 measured reflections	

### Refinement

**Table:**

Refinement on *F*^2^	Primary atom site location: structure-invariant direct methods
Least-squares matrix: full	Secondary atom site location: difference Fourier map
*R*[*F*^2^ > 2σ(*F*^2^)] = 0.048	Hydrogen site location: inferred from neighbouring sites
*wR*(*F*^2^) = 0.140	H-atom parameters constrained
*S* = 1.11	*w* = 1/[σ^2^(*F*_o_^2^) + (0.0713*P*)^2^ + 0.3322*P*] where *P* = (*F*_o_^2^ + 2*F*_c_^2^)/3
647 reflections	(Δ/σ)_max_ < 0.001
40 parameters	Δρ_max_ = 0.29 e Å^−^^3^
2 restraints	Δρ_min_ = −0.26 e Å^−^^3^

### Special details

**Table:**

Geometry. All esds (except the esd in the dihedral angle between two l.s. planes) are estimated using the full covariance matrix. The cell esds are taken into account individually in the estimation of esds in distances, angles and torsion angles; correlations between esds in cell parameters are only used when they are defined by crystal symmetry. An approximate (isotropic) treatment of cell esds is used for estimating esds involving l.s. planes.
Refinement. Refinement of F^2^ against ALL reflections. The weighted R-factor wR and goodness of fit S are based on F^2^, conventional R-factors R are based on F, with F set to zero for negative F^2^. The threshold expression of F^2^ > 2sigma(F^2^) is used only for calculating R-factors(gt) etc. and is not relevant to the choice of reflections for refinement. R-factors based on F^2^ are statistically about twice as large as those based on F, and R- factors based on ALL data will be even larger.

### Fractional atomic coordinates and isotropic or equivalent isotropic displacement parameters (Å^2^)

**Table:**

	*x*	*y*	*z*	*U*_iso_*/*U*_eq_	
N1	0.89057 (12)	0.5000	0.4836 (3)	0.0360 (5)	
H1A	0.8697	0.5000	0.3256	0.054*	
H1B	0.8394	0.5000	0.5653	0.054*	
C3	0.95255 (11)	0.3303 (2)	0.5542 (3)	0.0381 (4)	
H3A	0.9108	0.2210	0.5025	0.046*	
H3B	0.9736	0.3257	0.7246	0.046*	
O1	0.72210 (11)	0.5000	0.6756 (2)	0.0462 (5)	
O2	0.83814 (11)	0.5000	1.0091 (3)	0.0448 (5)	
C1	0.74651 (15)	0.5000	0.8955 (3)	0.0301 (5)	
C2	0.65942 (18)	0.5000	1.0283 (4)	0.0456 (6)	
H2A	0.5936	0.5000	0.9200	0.068*	
H2B	0.6649	0.3909	1.1257	0.068*	

### Atomic displacement parameters (Å^2^)

**Table:**

	*U*^11^	*U*^22^	*U*^33^	*U*^12^	*U*^13^	*U*^23^
N1	0.0222 (8)	0.0626 (12)	0.0242 (8)	0.000	0.0072 (6)	0.000
C3	0.0406 (9)	0.0424 (8)	0.0330 (8)	−0.0084 (6)	0.0113 (6)	0.0009 (6)
O1	0.0296 (8)	0.0834 (13)	0.0263 (8)	0.000	0.0075 (6)	0.000
O2	0.0319 (8)	0.0746 (12)	0.0277 (8)	0.000	0.0054 (6)	0.000
C1	0.0295 (10)	0.0355 (10)	0.0270 (9)	0.000	0.0093 (7)	0.000
C2	0.0397 (12)	0.0618 (15)	0.0410 (12)	0.000	0.0210 (10)	0.000

### Geometric parameters (Å, °)

**Table:**

N1—C3^i^	1.4770 (18)	C3—H3B	0.9700
N1—C3	1.4770 (18)	O1—C1	1.249 (2)
N1—H1A	0.9001	O2—C1	1.250 (2)
N1—H1B	0.9000	C1—C2	1.507 (3)
C3—C3^ii^	1.511 (3)	C2—H2A	0.9599
C3—H3A	0.9700	C2—H2B	0.9600
			
C3^i^—N1—C3	111.21 (15)	N1—C3—H3B	109.7
C3^i^—N1—H1A	108.5	C3^ii^—C3—H3B	109.7
C3—N1—H1A	108.5	H3A—C3—H3B	108.2
C3^i^—N1—H1B	106.6	O1—C1—O2	123.71 (18)
C3—N1—H1B	106.6	O1—C1—C2	117.29 (18)
H1A—N1—H1B	115.5	O2—C1—C2	119.00 (17)
N1—C3—C3^ii^	110.00 (10)	C1—C2—H2A	110.2
N1—C3—H3A	109.7	C1—C2—H2B	109.1
C3^ii^—C3—H3A	109.7	H2A—C2—H2B	109.5

Symmetry codes: (i) *x*, −*y*+1, *z*; (ii) −*x*+2, *y*, −*z*+1.

### Hydrogen-bond geometry (Å, °)

**Table:**

*D*—H···*A*	*D*—H	H···*A*	*D*···*A*	*D*—H···*A*
N1—H1A···O2^iii^	0.90	1.80	2.694 (2)	176.
N1—H1B···O1	0.90	1.79	2.680 (2)	170.

Symmetry codes: (iii) *x*, *y*, *z*−1.
